# A nomogram for bladder pain syndrome/interstitial cystitis based on netrin-1

**DOI:** 10.1007/s11255-021-03084-2

**Published:** 2021-12-13

**Authors:** Xiaojie Ang, Yufeng Jiang, Zongqiang Cai, Qi Zhou, Miao Li, Bin Zhang, Weiguo Chen, Li-Hua Chen, Xi Zhang

**Affiliations:** 1 Department of Urology, The First Affiliated Hospital of Soochow University, 188 Shizi Street, Suzhou, 215006 Jiangsu Province China; 2 Department of Urology, Kunshan Hospital of Traditional Chinese Medicine, No 189 Chao Yang Road, Kunshan, Jiangsu China; 3 Department of Nutrition and Food Hygiene, School of Public Health, Nantong University, Jiangsu, China

**Keywords:** Bladder pain syndrome, Interstitial cystitis, Netirn-1, Logistic regression model, Nomogram

## Abstract

**Purpose:**

This study aimed to combine plasma netrin-1 and clinical parameters to construct a diagnostic model for bladder pain syndrome/interstitial cystitis (BPS/IC).

**Methods:**

We analyzed the independent diagnostic value of netrin-1 and the correlation with clinical symptom scores of BPS/IC. Clinical parameters were selected using LASSO regression, and a multivariate logistic regression model based on netrin-1 was established, and then a nomogram of BPS/IC prevalence was constructed. The nomogram was evaluated using calibration curves, the C-index, and decision curve analysis (DCA). Finally, the model was validated using an internal validation method.

**Results:**

The area under the curve for the ability of netrin-1 to independently predict BPS/IC diagnosis was 0.858 (*p* < 0.001), with a sensitivity of 85% and specificity of 82%. The predicted nomogram included three variables: age, CD3 + /CD4 + T lymphocyte ratio, and netrin-1. The C-index of this nomogram was 0.882, and the predicted values were highly consistent with the actual results in the calibration curve. In addition, the internally validated C-index of 0.870 confirms the high reliability of the model. DCA results show that the net patient benefit of the netrin-1 combined with other clinical parameters was higher than that of the single netrin-1 model.

**Conclusion:**

Netrin-1 can be used as a diagnostic marker for BPS/IC and is associated with pain. The nomogram constructed by combining netrin-1 and clinical parameters was able to predict BPS/IC with great accuracy. In addition, Netrin-1 may also serve as a novel therapeutic target for BPS/IC.

## Introduction

Bladder pain syndrome/interstitial cystitis (BPS/IC) is a chronic condition of recurrent lower abdominal discomfort or pelvic pain associated with bladder filling in the absence of a urinary tract infection [1]. BPS/IC is associated with age, gender, and race, and the incidence of BPS/IC is 2–5 times higher in women than in men [2]. Currently, diagnosis of BPS/IC is based on clinical symptoms and staging based on cystoscopy to identify Hunner's ulcers in the bladder mucosa; an obvious drawback of such a test is its invasiveness [3]. In recent decades, potential biomarkers of BPS/IC have been sought using proteomics [4]. Nevertheless, no ideal single or combination of biomarkers has been identified.

Netrin-1 is a soluble protein secreted by basal plate cells; it was the first neural-specific factor identified in the netrin family [5, 6]. Netrin-1 is expressed in the nervous system and organs, such as the lung, pancreas, mammary gland, and intestinal epithelium [7]. Recent studies have revealed that netrin-1, a novel neuro-immune inflammatory factor, regulates the migration of inflammatory cells such as macrophages and the secretion of inflammatory factors [8]. In addition, netrin-1 has been associated with inflammatory pain in osteoarthritis and chronic pelvic pain associated with endometriosis [9, 10]. BPS/IC, a sterile inflammatory disease, has chronic pelvic pain as one of its main symptoms, and it is unclear whether netrin-1 is involved in the pathogenesis of BPS/IC.

In the present study, we aimed to investigate the diagnostic value of netrin-1 for BPS/IC and its clinical relevance. First, we examined netrin-1 expression in BPS/IC and analyzed the correlation between its expression and pain scores. Second, we constructed a novel nomogram prediction model based on netrin-1 and verified the reliability of the model.

## Methods

### Study design and participants

The Ethics Committee of the First Affiliated Hospital of Soochow University approved the study (approval number: 2020027), and all subjects provided written informed consent. The diagnosis of BPS/IC was based on the criteria established by the US National Institute of Diabetes and Digestive and Kidney Diseases [11]. Sixty women with non-ulcerated BPS/IC diagnosed by cystoscopy at the First Hospital of Soochow University between 03/2020 and 02/2021 were included in the BPS/IC group, and 60 healthy women undergoing check-ups at our hospital during the same period were selected as the control group. Healthy patients were defined as those with no history of disease other than diabetes or hypertension.

The inclusion criteria were as follows: (1) age between 18 and 70 years; (2) urination > 8 times during wakefulness, ≥ 2 episodes of nocturia per night, and ≥ 9 months duration of the condition; (3) bladder filling accompanied by discomfort in the pelvic area, perineum, lumbar region, or urethral vulva, with significant relief of pain or discomfort after urination; (4) bladder volume < 350 mL; (5) Hunner's ulcer or glomerular-like bleeding spot visible on cystoscopy, meeting the diagnostic criteria.

The exclusion criteria were as follows: (1) symptoms relieved with the application of antibiotics or antispasmodics; (2) association with diseases, such as urinary stones, urinary tract infections, or urinary tract tumors; (3) non-primary patients who received appropriate medication and had improved symptoms; (4) pregnant women and patients with allergies.

### Data collection

All subjects underwent testing for complete blood counts, C-reactive protein (CRP), and CD3 + CD4 + T lymphocyte ratio. 2 ml of blood was left to stand for 30 min and then centrifuged (3000 r/min for 10 min) and circulating levels of netrin-1 were evaluated with commercial ELISA kits (CSB-E11899h, Cusabio Biotech, Wuhan, China). The sensitivity of the ELISA kit was 7.81 pg/ml, with intra- and inter-batch coefficients of variation of less than 8% and 10%, respectively.

Participants’ information including age, height, weight, diabetes or hypertension was collected. Questionnaires about O'Leary-Sant scale problem index (ICPI), the interstitial cystitis symptom index (ICSI), and a visual analog scale (VAS) were administered to patients in the BPS/IC group.

### Logistic regression modeling

The “glmnet” package in R 4.0.0 software was used to select clinical characteristics using LASSO regression for 120 subjects with regard to age, BMI, hypertension, diabetes, menopause, CRP level, neutrophil-to-lymphocyte ratio (NLR), CD3 + CD4 + T lymphocyte ratio, and netrin-1 level. LASSO regression was used to select the most important features to avoid overfitting or overcomplicating the model [12, 13]. The independent variables obtained from the LASSO regression screening were incorporate into the multivariate logistic regression model [14].

### Construction and evaluation of nomograms

The “rms” package in the R 4.0.0 software was used to create nomograms based on the contribution of each variable in the logistic regression model to the outcome variables [15]. After plotting the nomogram, the consistency C-index of the model was calculated, and the nomogram was evaluated using the graphical calibration method. The receiver operating characteristic (ROC) curve for a single netrin-1 diagnosis and decision curve analysis (DCA) for netrin-1 combined with other clinical parameters were plotted using the “pROC” and “rmda” packages in R 4.0.0 software, respectively, to compare the advantages and disadvantages of the models [16].

### Statistical methods

Statistical analysis was performed using the R software (Version 4.0.0; https://www.R-project.org). The mean ± standard deviation (*x* ± *s*) was used for those with normal distribution, while the median and interquartile range were used for skewed distribution. The ROC of each parameter was plotted using the R software package “pROC” to determine the diagnostic value, and the correlations between netrin-1 and BPS/IC were analyzed using Spearman's rank correlations. Clinical characteristics were then selected using LASSO regression, logistic regression models were constructed and Nomograms were plotted. The accuracy of the model was assessed using C-index, calibration curves, ROC and DCA. Finally, the accuracy of the prediction model was verified by calculating the C-index using an internal validation method (1000 Bootstrap self-sampling) [17]. Differences were considered statistically significant at *P* < 0.05.

## Results

### Characteristics of the study participants

The mean ages of the BPS/IC and control groups were 50.30 ± 10.34 years (26–68 years) and 51.53 ± 11.11 years (25–69 years). Cystoscopic Hunner’s ulcers were not seen in any patient in the BPS/IC group. There were no significant differences between groups with regard to age, BMI, CRP, or NLR. The mean values of CD3 + CD4 + T lymphocyte ratio were 41.48 ± 6.18% and 37.38 ± 2.71% in the two groups, respectively (*P* < 0.001, Table 1).

**Table 1:** Comparison of various clinical characteristics between the two groups

Characteristics	Group		Significance test	
	BPS/IC (*n* = 60)	Control (*n* = 60)	*t*/χ^2^	*P-*value
Age (years)	50.30 ± 10.34	51.53 ± 11.11	−0.630	0.530
BMI (kg·m^−2^)	22.86 ± 3.11	22.72 ± 2.16	0.280	0.780
CD3 + CD4 + (%)	41.48 ± 6.18	37.38 ± 2.71	4.704	< 0.001
CRP (mg/L)	0.99 ± 1.03	0.97 ± 0.61	0.121	0.904
NLR	2.25 ± 1.03	2.21 ± 0.73	0.248	0.805
Comorbidities, number of case (%)				
Hypertension	17 (28.3)	21 (35.0)	0.616	0.432^a^
Diabetes	17 (28.3)	16 (26.7)	0.042	0.838^a^
Pausimenia	32 (53.3)	32 (53.3)	0.000	1.000^a^

Analysis by paired *t* tests

*BMI* body mass index; *CRP* C-reactive protein; *NLR* neutrophil-to-lymphocyte ratio

^a^Cardinality test (Monte Carlo significance)

**Fig. 1:**
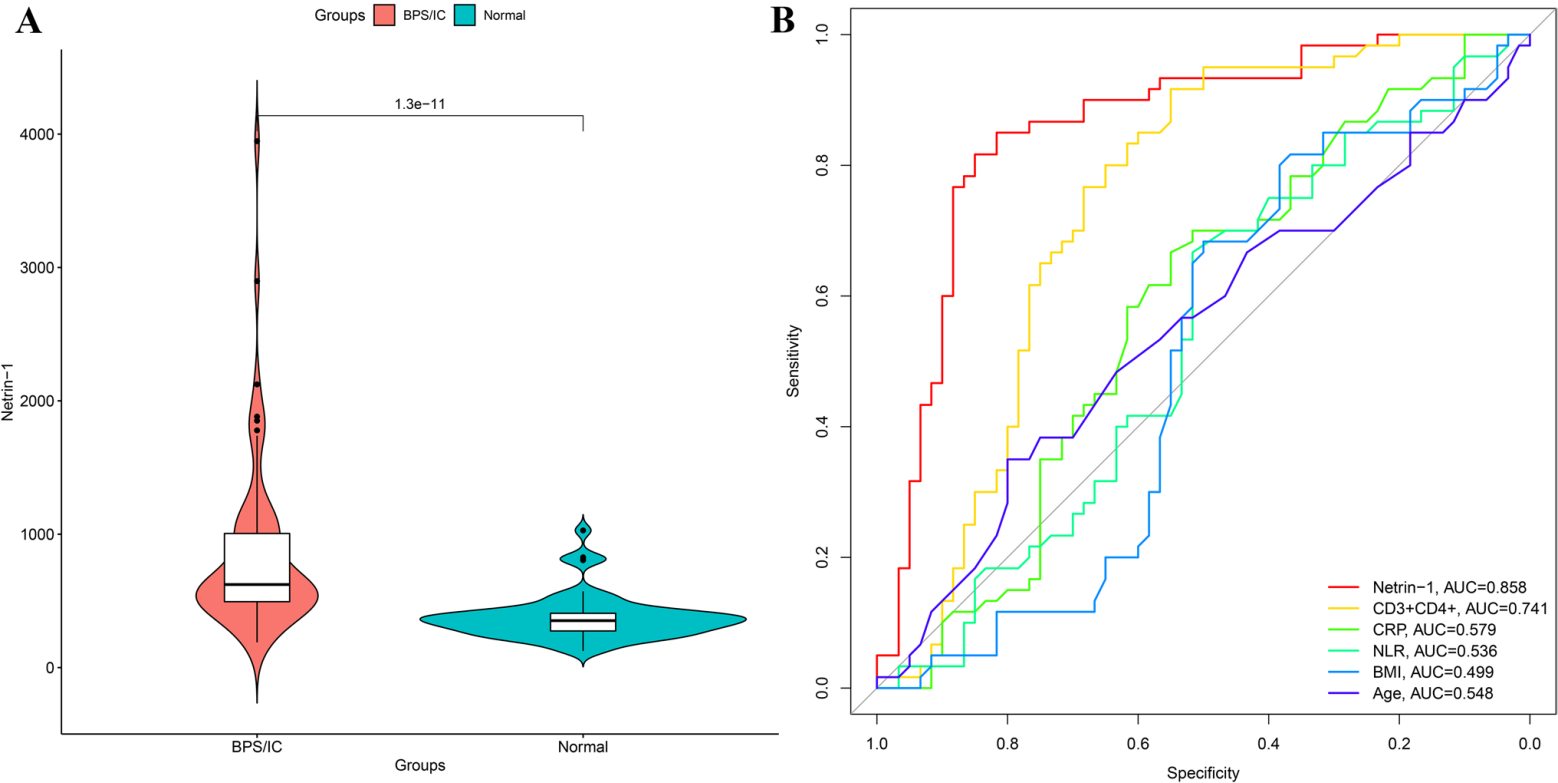
**A** Violin plot of plasma netrin-1 levels in the BPS/IC and control groups. Plasma netrin-1 levels in the BPS/IC group were significantly higher than those in the control group, *P* < 0.001. **B** Comparison of the diagnostic efficacy of various clinical parameters for BPS/IC, in which netrin-1 and CD3 + CD4 + T lymphocyte ratio had good diagnostic efficacy, while age, BMI, CRP, and NLR had relatively poor diagnostic value

The plasma netrin-1 levels in the BPS/IC and control groups were 847.30 ± 654.55 pg/ml and 373.00 ± 167.51 pg/ml, respectively (*P* < 0.001, Fig. 1A). The area under the ROC curve (AUC) for the ability of netrin-1 level to independently predict a diagnosis of BPS/IC was 0.858 (95% CI: 0.788 to 0.929, *P* < 0.001), with better diagnostic efficacy than other clinical parameters including age, BMI, CRP, and NLR (Fig. 1B). The optimal cut-off value was 443.46 pg/ml (the sensitivity was 85%, and the specificity was 82%).

### Correlation between netrin-1 and clinical symptom scores

**Fig. 2:**
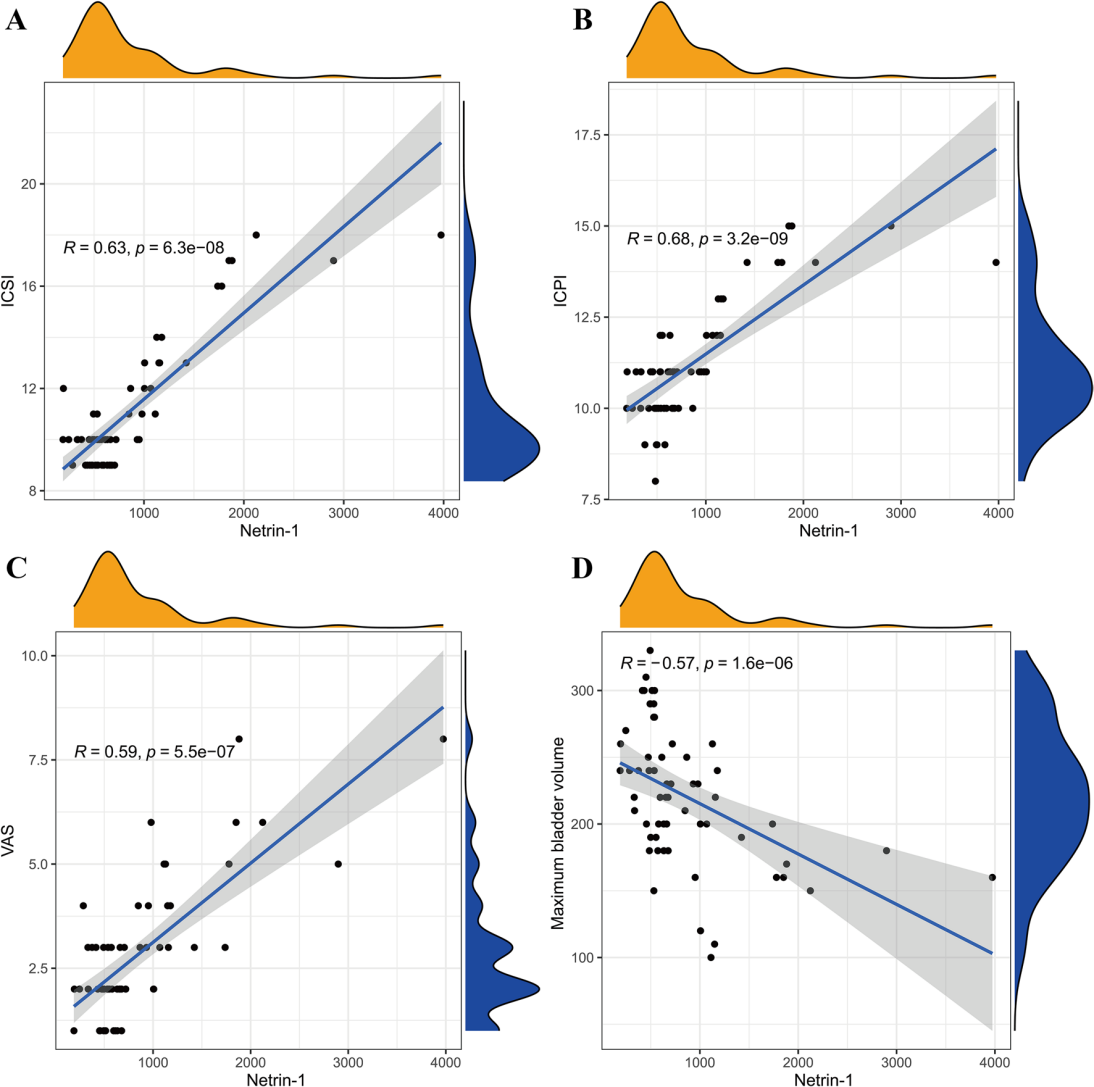
Correlation analysis between netrin-1 and BPS/IC clinical symptom scores. **A** Netrin-1 correlated with ICSI with a coefficient of 0.63, a moderately positive correlation; **B** Netrin-1 correlated with ICPI with a coefficient of 0.68, a moderately positive correlation; **C** Netrin-1 correlated with VAS with a coefficient of 0.59, a moderate positive correlation; **D** Netrin-1 correlated with maximum bladder capacity with a coefficient of -0.57, a moderately negative correlation

The correlation coefficients between plasma netrin-1 levels and patient ICSI, ICPI, VAS score, and maximum bladder capacity in BPS/IC patients were 0.63, 0.68, 0.59, and −0.57, respectively. There were moderately positive correlations between plasma netrin-1 levels and patient ICSI, ICPI, and VAS in BPS/IC patients and a moderate negative correlation with maximum bladder capacity (Fig. 2A–D).

### Logistic regression model

**Fig. 3:**
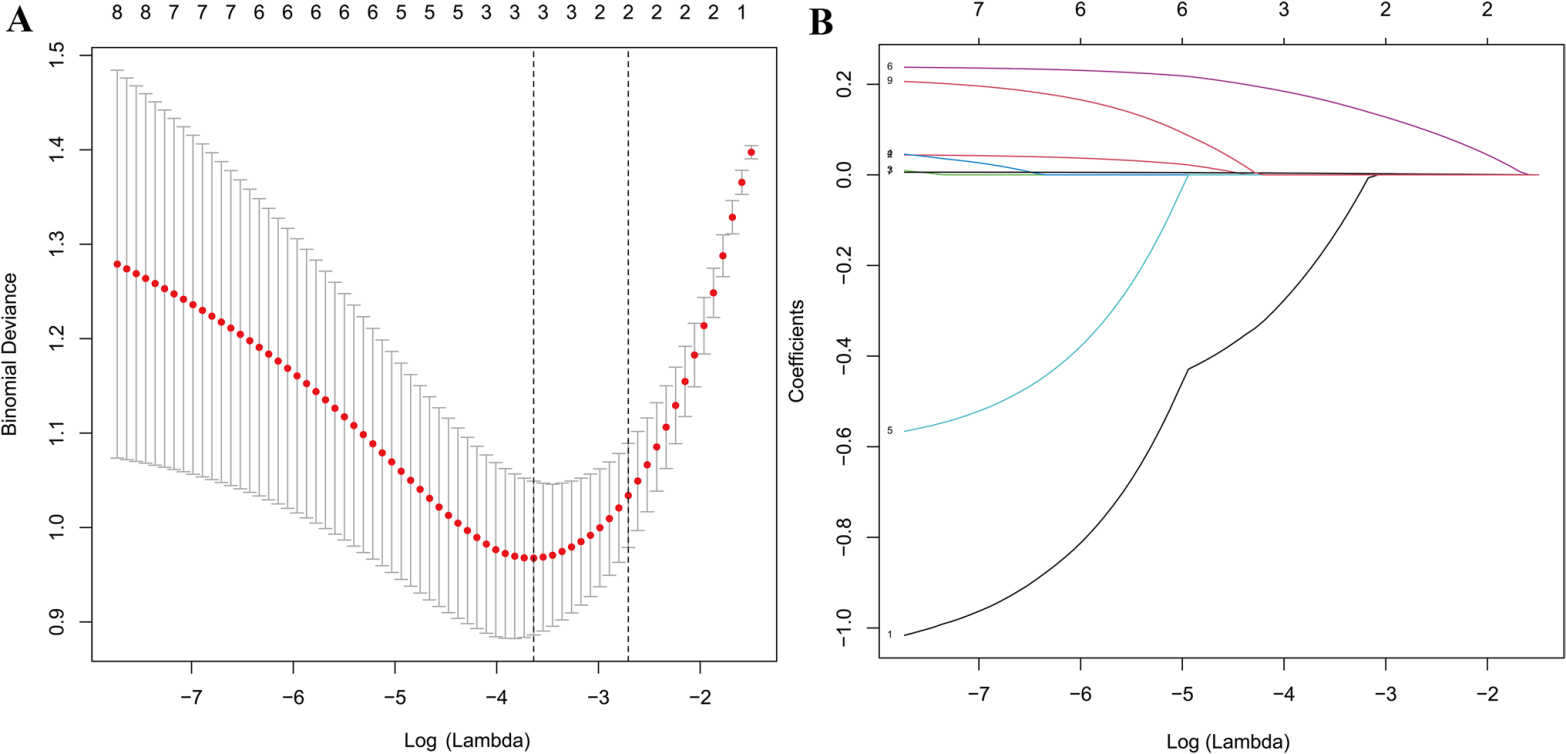
Screening predictors for LASSO regression analysis. **A** The vertical line plotted at the optimal parameter lambda = 0.0182 with three selected variables. **B** The plot of each clinical characteristic coefficient versus log(lambda) by adjusting the parameter lambda value

**Fig. 4:**
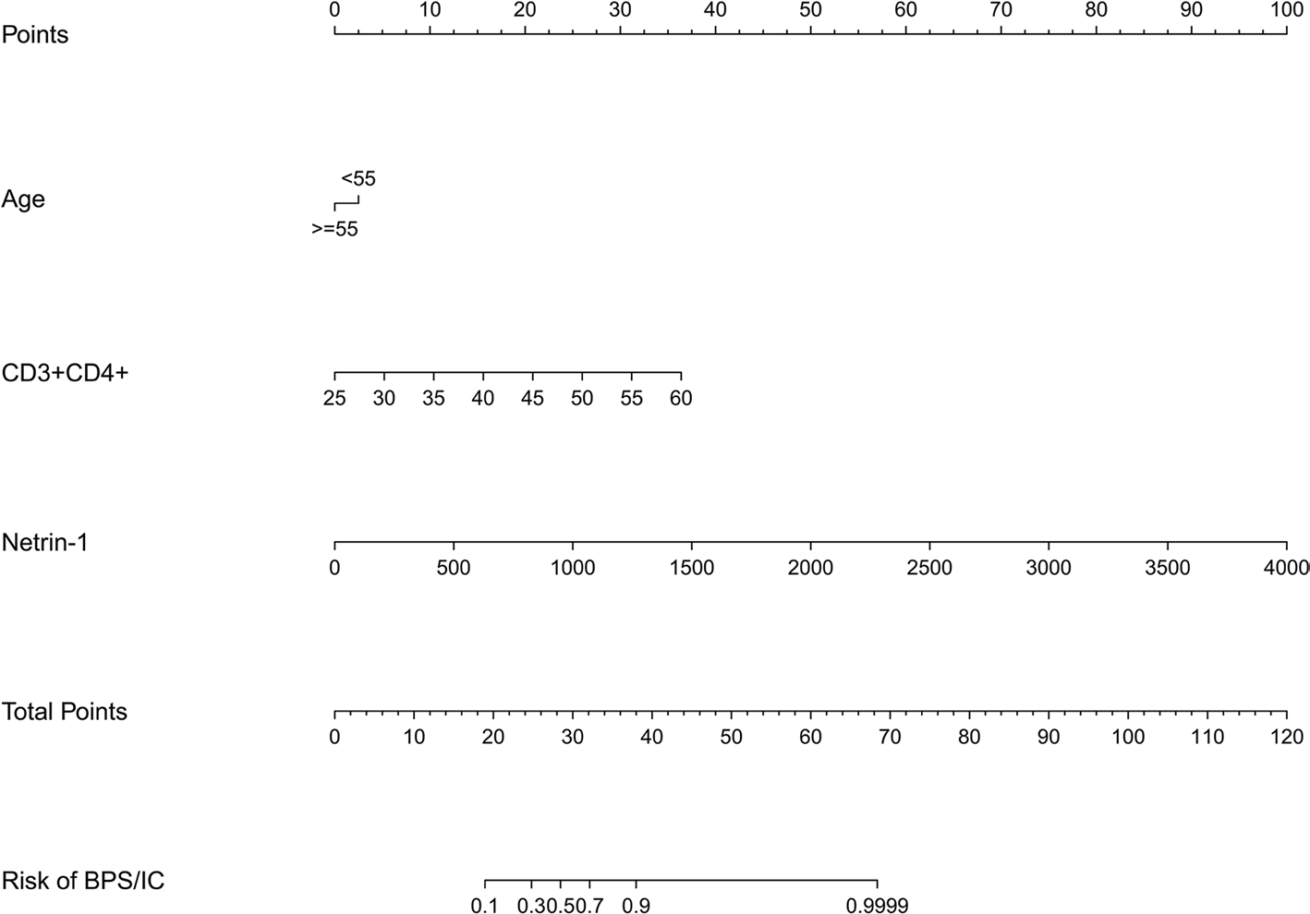
Nomogram of the BPS/IC diagnostic prediction model for netrin-1 combined with clinical parameters. Note: For three variable axes (age, CD3 + CD4 + T lymphocyte ratio, and netrin-1 level), the corresponding points on the axes correspond to different scores. The scores of each predictive variable are summed to obtain a total score. Different total scores correspond to different probabilities of BPS/IC prevalence

Screening the clinical parameters using LASSO regression revealed that three independent variables were included in the model: age (older than 55 years), CD3 + CD4 + T lymphocyte ratio, and netrin-1 levels (Fig. 3). A multivariate logistic regression model was developed based on these variables (Table 2). CD3 + CD4 + T lymphocyte ratio and netrin-1 were independent risk factors (*P* < 0.001). For three variable axes (age, CD3 + CD4 + T lymphocyte ratio, and netrin-1 level), the corresponding points on the axes correspond to different scores. The scores of each predictive variable are summed to obtain a total score. Different total scores correspond to different probabilities of BPS/IC incidence (Fig. 4).

**Table 2:** BPS/IC multivariate logistic regression analysis

Intercept and variable	Prediction model		
	β	Odds ratio (95% CI)	*P*-value
Intercept	−11.9891	0.000 (0.000–0.001)	< 0.001
Age	−0.5749	0.563 (0.204–1.485)	0.252
CD3 + CD4 + (%)	0.2399	1.271 (1.114–1.494)	0.001
Netrin-1	0.0058	1.006 (1.003–1.009)	< 0.001

Note: β is the regression coefficient

### Construction and evaluation of nomograms

**Fig. 5:**
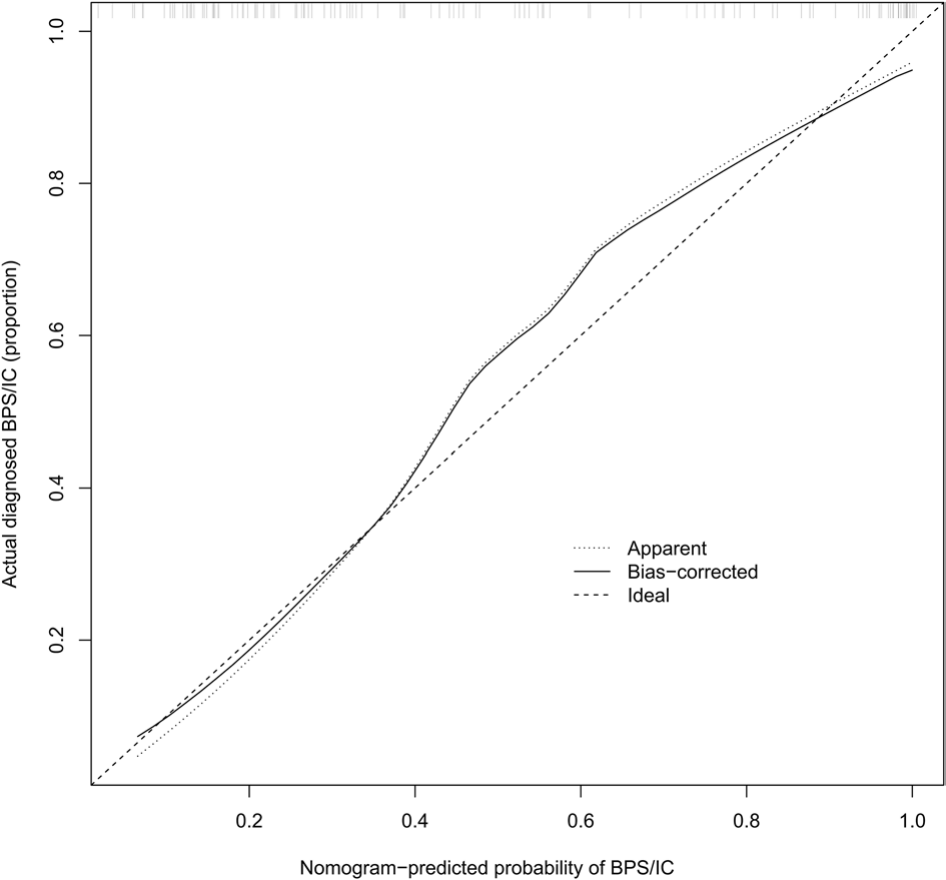
Calibration curve of the BPS/IC nomogram model for netrin-1 combined with other clinical parameters. Note: The *x*-axis is the predicted BPS/IC occurrence probability of the nomotube model, and the *y*-axis is the actual BPS/IC occurrence rate. The diagonal dashed line represents the ideal model prediction performance; the solid line represents the prediction performance of the nomotube model in this study. The closer it is to the diagonal dashed line, the better the prediction performance

The calibration curve showed that the predicted incidence of BPS/IC was close to the actual results (the C-index of the model was 0.882, which is a high predictive value) (Fig. 5). The C-index of the model was 0.870 after validation of the diagnostic prediction model using internal validation, and the model has excellent predictive performance.

**Fig. 6:**
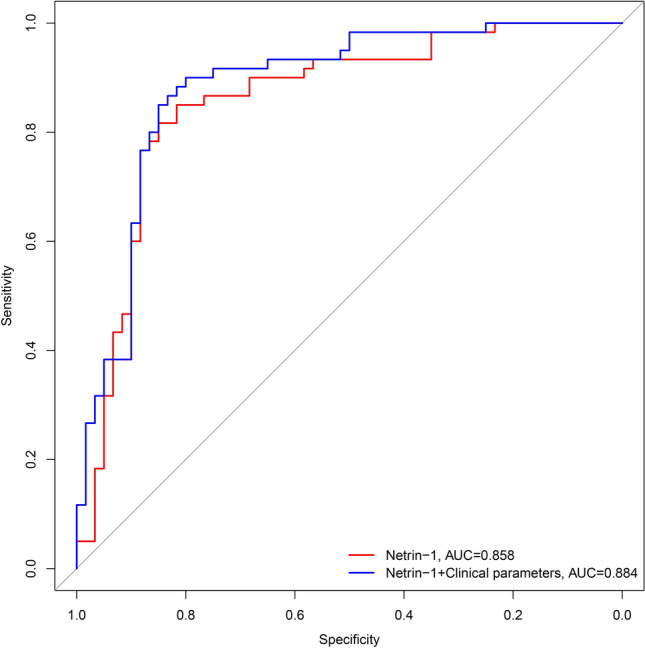
ROC for the diagnosis of BPS/IC with the nomogram model for single netrin-1 and netrin-1 combined with other clinical parameters. Note: The two curved diagonal lines in the figure represent two different clinical diagnostic models (see legend label). Netrin-1 alone diagnosed BPS/IC with an AUC at ROC of 0.858 (95% CI: 0.788–0.929, *P* < 0.001) and the nomogram model with netrin-1 combined with other clinical parameters had an AUC of the ROC of 0.884 (95% CI: 0.821–0.946, *P* < 0.001). 95% CI: 0.821–0.946, *P* < 0.001). The diagnostic efficacy of the nomogram model with netrin-1 combined with other clinical parameters was slightly better than that of netrin-1 alone

The AUC of ROC for the ability of netrin-1 level to independently predict a diagnosis of BPS/IC was 0.858 (95% CI: 0.788–0.929, *P* < 0.001), and the AUC of ROC for the nomogram model of netrin-1 combined with other clinical parameters was 0.884 (95% CI: 0.821–0.946, *P* < 0.001), with a sensitivity of 80% and specificity of 90%. The diagnostic efficacy of the nomogram model of netrin-1 combined with other clinical parameters was slightly better than that of netrin-1 alone (Fig. 6).

**Fig. 7:**
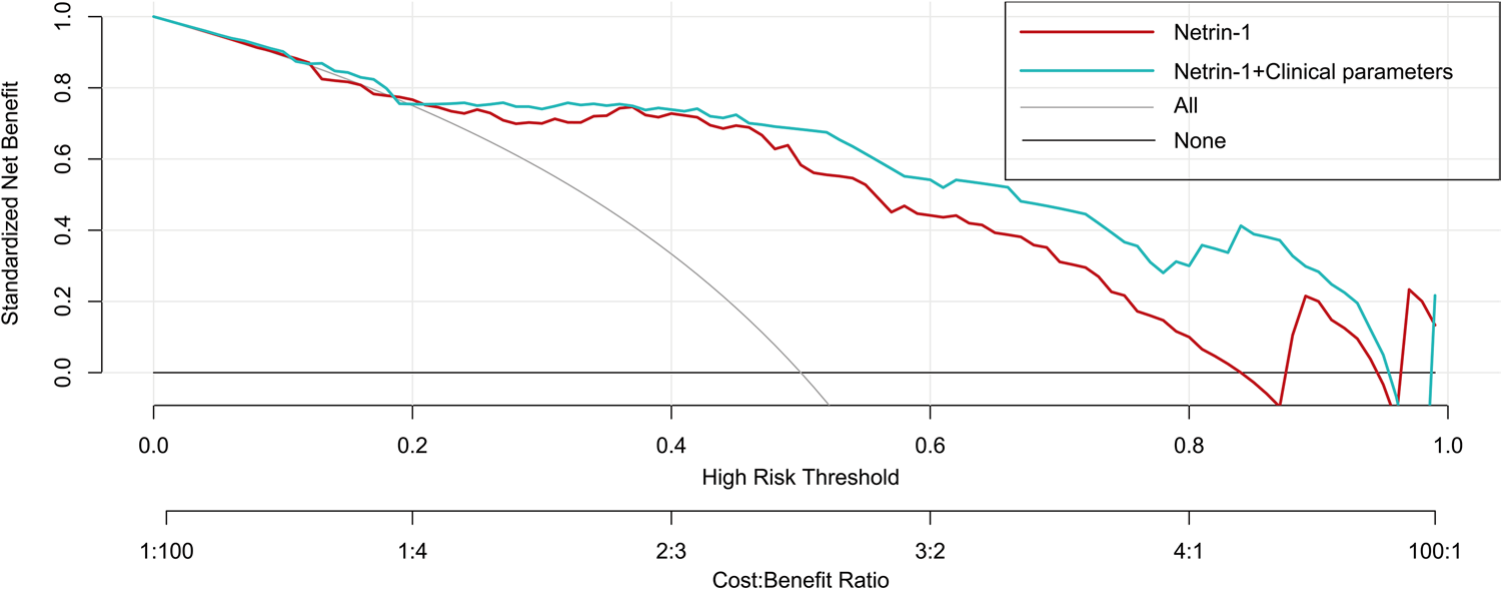
DCA for the diagnosis of BPS/IC with a single netrin-1 and netrin-1 combined with other clinical parameters of the nomogram model. Note: The *x*-axis is the threshold probability, the y-axis is the net benefit normalized by prevalence, and the two curved sloping lines in the figure represent two different clinical diagnostic models (see legend). The net benefit for patients in the diagnostic prediction model with netrin-1 combined with other clinical parameters is significantly higher than the net benefit for patients diagnosed with netrin-1 alone

Decision curve analysis (DCA) showed that the threshold probability range for patients was 10–85% for netrin-1’s ability to independently diagnose BPS/IC and 3–96% for the diagnostic model with netrin-1 combined with other clinical parameters. The net benefit to patients when using the diagnostic prediction model with netrin-1 combined with other clinical parameters was significantly better than that of netrin-1 alone (Fig. 7).

## Discussion

In this study, we made two critical clinical findings. First, netrin-1 can be used as a biomarker for BPS/IC. Second, the diagnostic model of netrin-1 combined with clinical parameters has high predictive value. To our knowledge, this study is the first report to describe a diagnostic model for BPS/IC using netrin-1.

BPS/IC is a chronic functional disorder characterized by hypersensitivity reactions in the lower urinary tract [18]. Approximately 8 million women worldwide may currently suffer from BPS/IC, and perhaps many more remain undiagnosed [19]. Therefore, the early, rapid, and accurate diagnosis of BPS/IC needs to be addressed. Many markers in BPS/IC serum and urine have been described in previous studies; however, all have shortcomings. The sensitivity of GP51 in the urine of BPS/IC patients is too low. Anti-proliferative factor (APF) is the most sensitive and specific diagnostic marker for BPS/IC with a sensitivity of 94% and specificity of 95%; however, it is not yet widely used in clinical practice due to the complexity of the APF assay technique. Nerve growth factor in urine is simple to measure; however, it is not sufficiently specific, and levels may be increased in the presence of urinary stones, urinary tract infection, or bladder outlet obstruction [20–22].

Serum and plasma specimens are less susceptible to contamination than urine specimens and better reflect the systemic nature of the disease. It has been shown that serum levels of serum pro-inflammatory cytokines (interleukins 1-beta and 6 and tumor necrosis factor-alpha) and chemokines (interleukin 8) are significantly higher in patients with BPS/IC than in controls; However, these are less sensitive and specific [23, 24]. There is a growing consensus that BPS/IC is associated with neuro-immune inflammation, that pain associated with BPS/IC is considered a form of neuropathic pain. Although growing evidence showed that the novel neuro-immune inflammatory factor netrin-1 regulated pain, there are no relevant studies of netrin-1 in BPS/IC [25, 26]. The potential biological relevant role of netrin-1 with BPS/IC and easily measurable by commercial available kit build our rationale to study netrin-1 levels in BPS/IC serum. Our results showed that the AUC of the ROC for was 0.858 (sensitivity of 85%, specificity of 82%) for netrin-1 to diagnose of BPS/IC. Netrin-1 was also correlated significantly with ICSI, ICPI scores, VAS scores, and maximum bladder capacity. As ICSI and ICPI scores increased and symptoms worsened, netrin-1 levels increased accordingly. Therefore, netrin-1 could be a potential molecular diagnostic marker for BPS/IC, and elevated netrin-1 in plasma of women with BPS/IC is positively correlated with pain scores. This may provide a novel target and therapeutic idea for the management of BPS/IC, and the related mechanism deserves further investigation.

Logistic regression models are potent tools often used in clinical outcome studies. When applied uncritically, these models may generate results that do not fit well with the available data set or that do not accurately predict the outcome of a study. Therefore, it is necessary to perform LASSO regression to screen variables before building a logistic regression model to avoid poor fitting or overfitting [27, 28]. In the present study, we constructed a rapid and straightforward clinical diagnostic prediction model with an AUC of 0.884 under ROC, a sensitivity of 80%, a specificity of 90%, and a DCA with a threshold probability interval 3–96%. The internal validation results suggest that the model has a discriminant C-index of 0.870 and excellent model predictive performance. The AUC of the ROC improved slightly when comparing netrin-1 alone with netrin-1 combined with other clinical parameters for prediction of BPS/IC. However, DCA showed a more significant difference, with a higher net benefit to patients from the diagnostic prediction model of netrin-1 combined with other clinical indicators. DCA is a simple method for evaluating clinical predictive models, diagnostic tests and molecular biomarkers [29]. In contrast to traditional performance measures, such as sensitivity, specificity and AUC, which failed to take into account the clinical utility of a particular model, DCA plots net benefit at a range of clinically reasonable risk thresholds, which can help to assess the utility of models for decision-making [30].

## Conclusion

Overall, netrin-1 is a relatively accurate and valuable marker, and the nomogram model constructed based on netrin-1 can predict BPS/IC with great accuracy to assist physicians in making better clinical decisions. And the greater significance of this study is that it provides a new direction for research into the etiology and pathophysiology of BPS/IC. However, large data samples and multi-centre studies are needed to validate our findings.

## Data Availability

The datasets generated and analysed during the current study are not publicly available due to protect patient privacy.
